# Exfoliated black phosphorous-mediated CuAAC chemistry for organic and macromolecular synthesis under white LED and near-IR irradiation

**DOI:** 10.3762/bjoc.17.164

**Published:** 2021-09-23

**Authors:** Azra Kocaarslan, Zafer Eroglu, Önder Metin, Yusuf Yagci

**Affiliations:** 1 Department of Chemistry, Istanbul Technical University, Maslak, 34469 Istanbul, Turkey; 2 Department of Chemistry, Koç University, Sarıyer, 34450, Istanbul, Turkey; 3 Department of Nanoscience and Nanoengineering, Atatürk University, 25240 Erzurum, Turkey; 4 King Abdulaziz University, Faculty of Science, Chemistry Department, 21589 Jeddah, Saudi Arabia

**Keywords:** black phosphorus, click chemistry, heterogeneous photocatalyst, near infrared, phosphorene

## Abstract

The development of long-wavelength photoinduced copper-catalyzed azide–alkyne click (CuAAC) reaction routes is attractive for organic and polymer chemistry. In this study, we present a novel synthetic methodology for the photoinduced CuAAC reaction utilizing exfoliated two-dimensional (2D) few-layer black phosphorus nanosheets (BPNs) as photocatalysts under white LED and near-IR (NIR) light irradiation. Upon irradiation, BPNs generated excited electrons and holes on its conduction (CB) and valence band (VB), respectively. The excited electrons thus formed were then transferred to the Cu^II^ ions to produce active Cu^I^ catalysts. The ability of BPNs to initiate the CuAAC reaction was investigated by studying the reaction between various low molar mass alkyne and azide derivatives under both white LED and NIR light irradiation. Due to its deeper penetration of NIR light, the possibility of synthesizing different macromolecular structures such as functional polymers, cross-linked networks and block copolymer has also been demonstrated. The structural and molecular properties of the intermediates and final products were evaluated by spectral and chromatographic analyses.

## Introduction

For the last decade, click chemistry has been recognized as an indispensable part of synthetic chemistry due to its easiness of application, efficiency to produce the targeted products with very high yields and little or no byproducts under a variety of conditions, and high interconnected group tolerance. Since the introduction of click chemistry by Sharpless [1–2] and Mendal [3], many studies have been dedicated to better understanding of the concept and expanding its scope to be applied in various fields of chemistry including bioconjugation [4], drug discovery [5], materials science [6–9] and so on [10]. The development of the use of light in click chemistry has set a milestone as a new and effective method for the synthesis of macromolecules [11]. Initiation of this reaction photocatalytically provides many advantages for the synthetic methodologies including bioconjugation, labeling, surface functionalization, dendrimer synthesis, polymer synthesis, and polymer modification by adding spatial and temporal control [12–13].

In recent years, heterogeneous photocatalysts have been performed in many photosynthetic reactions since they provide a more reasonable and easy way to synthesize the targeted products compared to the classical homogenous photocatalysts. In this respect, 2D materials offer great potential due to converting the inexhaustible energy of sunlight into chemical and electrical energy along with having a less environmental impact. After the discovery of the photocatalytic effect of 2D materials under UV light [14–15] the heterogeneous photocatalysts have been successfully applied in both small- and large-scale synthesis such as organic reactions [16–17], free radical polymerization (FRP) [18–20], controlled radical polymerization (CRP) [21–22], CuAAC chemistry [23–25], and thiol–ene chemistry [26–27]. However, most of the conventional 2D materials have a wide bandgap that requires UV light irradiation for their activation. Since 94% of the rays from the sun are not sufficient to activate these conventional semiconductor materials, many strategies have been proposed to design photocatalysts that can harvest in a wide spectrum of sunlight, especially in the NIR region [28–29]. In particular, the development of new photocatalyst systems that absorb the incident light from the sun at much longer wavelengths have aroused widespread interest [30–33]. However, the most of the NIR photocatalysts applied exhibit relatively low catalytic efficiency due to their low absorption characteristics and require complicated synthetic procedures. In this respect, it is worth to mention that elemental 2D materials with a proper bandgap and charge mobilities have been shown to act as photocatalysts in several reactions [34–35]. Exfoliated black phosphorus (BP), the most stable allotrope of phosphorus, has been shown as a highly efficient photocatalyst possessing superior features in many respects [36–37]. BP, a vital semiconductor 2D material with excellent physicochemical properties such as high carrier mobility, tunable optical absorption, and novel electronic band structure, fills the gap between graphene and wide bandgap semiconductors [35,38]. Furthermore, BP shows a layer thickness tunable bandgap ranging between 0.3 and 2.1 eV. Therefore, BPNs can efficiently be applied as a photoredox catalyst with broadband solar absorption [34,38–40].

The use of 2D materials for the photoinitiated electron transfer reactions with Cu^II^ catalysts for the photoinduced atom transfer radical polymerization (ATRP) and CuAAC reactions prompted us to develop a new photoredox system that works under NIR irradiation for the CuAAC reaction. In this work, we report a new synthetic strategy to the photochemical reduction of Cu^II^ to Cu^I^ for the CuAAC reaction using BPNs as the photo-initiator under NIR light.

## Results and Discussion

**Figure 1:**
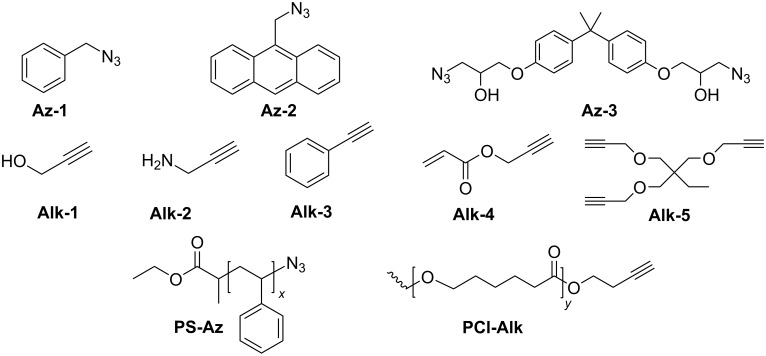
Structures of azide and alkyne functional molecules and polymers used in the photoinduced CuAAC reactions.

The detailed preparation and characterization of the initial BP crystals and BPNs were previously reported [40]. BPNs were tested as NIR photoinitiator for the CuAAC reactions of low molar mass compounds and polymers possessing antagonist azide and alkyne functionalities (Figure 1).

**Figure 2:**
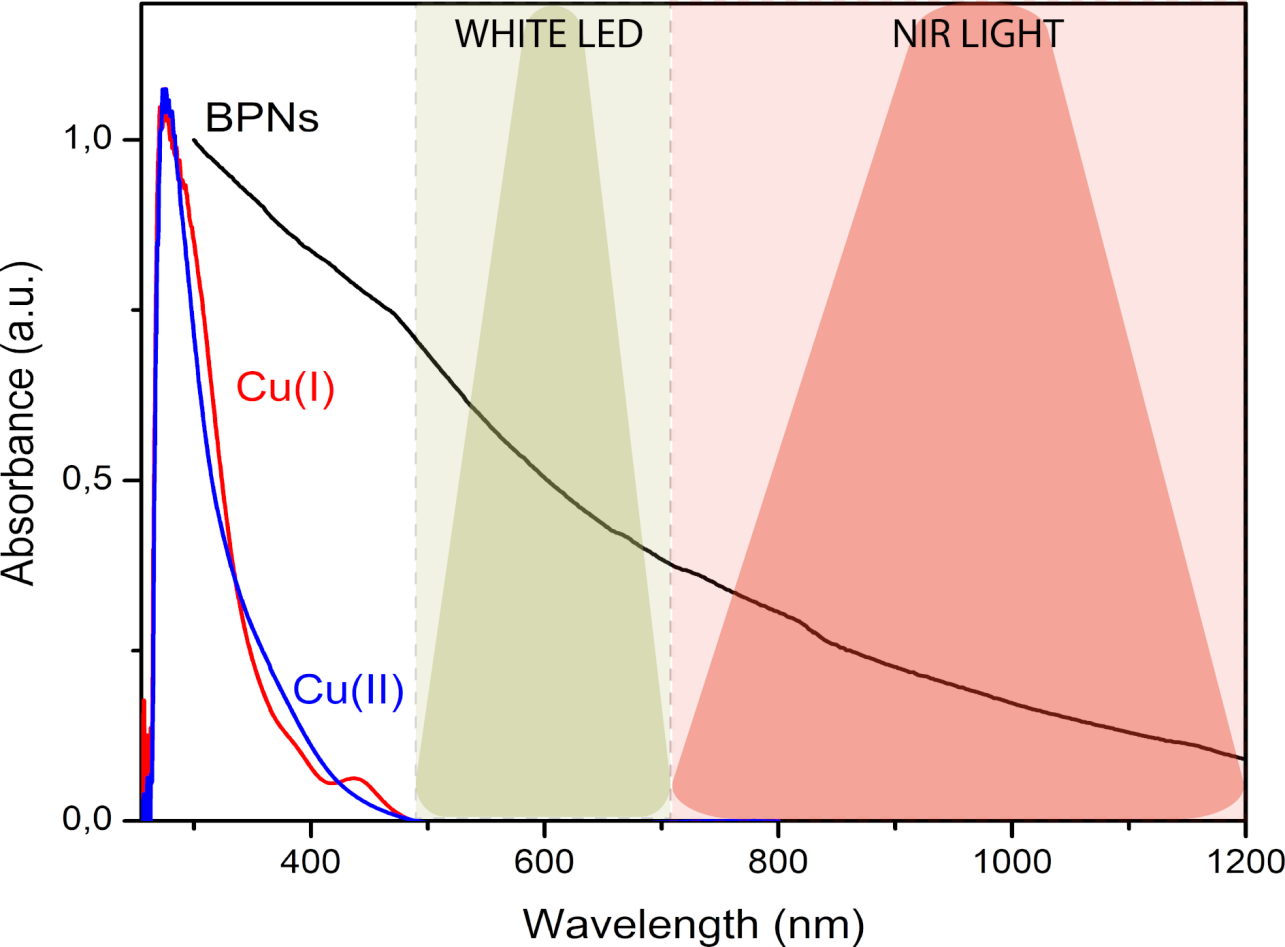
UV–vis spectra of Cu^I^Cl, Cu^II^Cl_2_ and BPNs.

The optical absorption spectra of BPNs, copper(I) chloride (Cu^I^Cl, 0.05 mmol) and copper(II) chloride (Cu^II^Cl_2,_ 0.05 mmol) are shown in Figure 2. As can be seen, the BPNs displayed an excellent wide range of light absorption region up to 1000 nm. In this regard, BPNs are the only light absorbing component in the NIR region where Cu^II^ is completely transparent.

**Figure 3:**
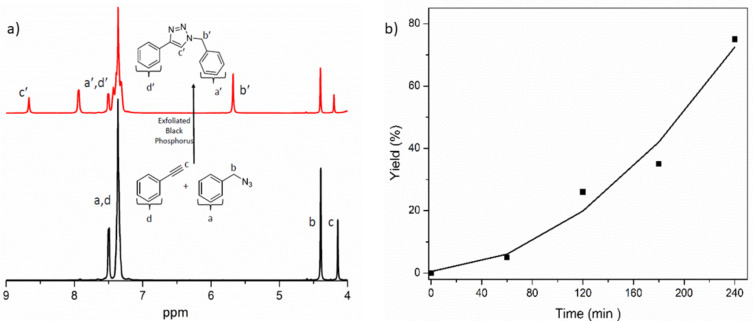
a) ^1^H NMR spectra of the model reaction between benzyl azide (**Az-1**) and phenylacetylene (**Alk-3**) before (black) and after (red) irradiation. b) Conversion-time plot as measured by ^1^H NMR spectroscopy through integration of the acetylene proton around 4.42 ppm.

Initially, the model reaction between benzyl azide (**Az-1**) and phenylacetylene (**Alk-3**) in the presence of copper(II) chloride/ *N*,*N*,*N*’,*N*’,*N*’’-pentamethyldiethylenetriamine (Cu^II^Cl_2_/PMDETA) and exfoliated BPNs under the white LED irradiation was performed (Figure 3). The reaction was followed by ^1^H NMR spectroscopy during the click process. The decrease of the acetylene proton at 4.42 ppm and appearance of the new signal at 8.67 ppm corresponding to the triazole moiety confirmed successful click reaction under white LED exposure conditions after 4 h (Figure 3a). Kinetic studies conducted by ^1^H NMR analysis confirmed that the click reaction between benzyl azide and phenylacetylene resulted in almost complete conversion within 4 h white LED irradiation (Figure 3b). In this connection, it should be pointed out that the reaction proceeds also in dark almost at the same rate (Supporting Information File 1, Figure S4). This is an expected observation because there is no back reaction to reform Cu(II). Similar observations were reported by the other photoinduced CuAAC reactions [41].

In order to demonstrate the functional group tolerance, the extent of the reaction was investigated on various alkyne groups using benzyl azide under both white LED and NIR light irradiation. The results presented in Table 1 revealed that NIR-light-triggered click reactions produced the corresponding products with slightly higher yields favored by the higher penetration of NIR light to the reaction media containing heterogeneously dispersed BPNs. Compared with propargyl alcohol (**Alk-1**) and propargyl acrylate (**Alk-4**), the rate of clicking slightly decreased in the case of propargylamine (**Alk-2**), but still gave high yields. Therefore, it can be concluded that **Alk-2** and **Alk-1** exhibit relatively lower efficiency probably due to the additional coordination of the Cu^I^ catalyst. Notably, the reaction with **Alk-4** gave higher yields with both light sources.

**Table 1:** Photoinduced CuAAC between benzyl azide and various alkynes^a^ using exfoliated BPNs^b^ in DMSO-*d*_6_ = 1 g/L.

			White LED	NIR Light
Run	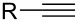	Product	Yield^c^	Yield^c^

1	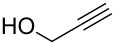	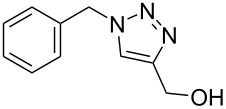	43	98
2	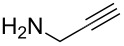	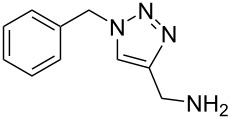	65	69
3	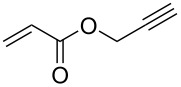	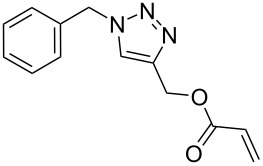	90	95

^a^All reactions were carried out in a NMR tube in a room temperature in the presence of Cu^II^Cl_2_/PMDETA. ^b^Reaction time = 4 h. ^c^Conversions were determined by ^1^H NMR spectroscopy.

**Scheme 1:**
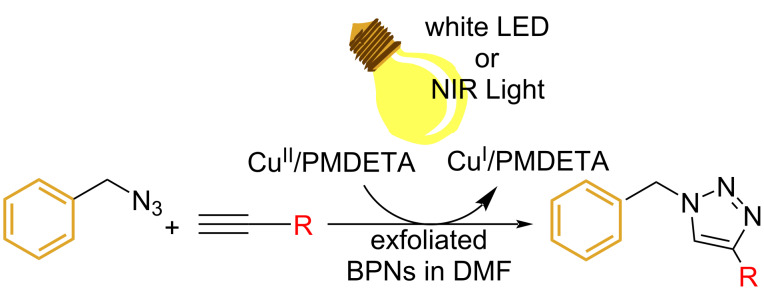
Proposed mechanism for photoinduced CuAAC reaction using exfoliated BPNs.

In the light of previous studies, a photoinduced electron transfer mechanism presented in Scheme 1 can be proposed. Upon the light irradiation, BPNs absorb the light and generate a single electron which was transferred from the conduction band to the Cu^II^ complex to form Cu^I^ capable of catalyzing the click reaction in a conventional manner.

**Figure 4:**
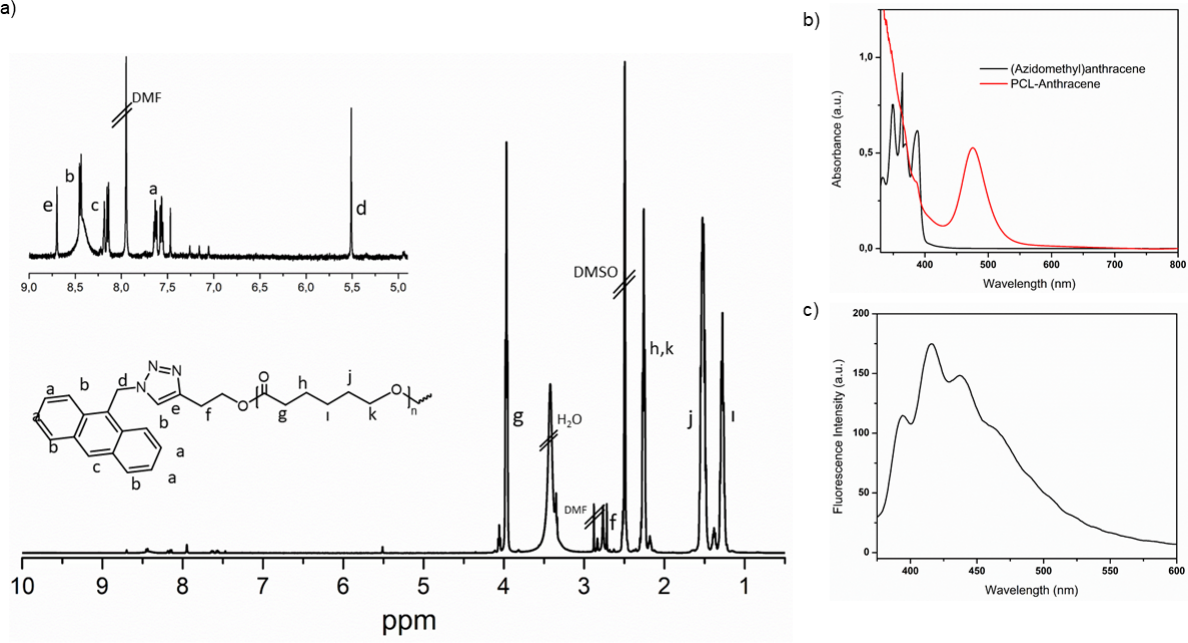
a) ^1^H NMR spectrum of chain end modified **PCL-Anth**; b) UV–vis spectra of (azidomethyl)anthracene (black) and **PCL-Anth** (red); c) fluorescence emission spectrum of **PCL-Anth**.

The applicability of the described click reaction to synthetic polymer chemistry was also demonstrated. For this purpose, polymer functionalization by using alkyne functional poly(ε-caprolactone) (**PCL-Alk**) and 9-(azidomethyl)anthracene (**Az-2**) as click components was investigated. The detailed ^1^H NMR spectrum of the resulting anthracene functional polymer (**PCL-Anth**) exhibited the characteristic signals of triazole and benzylic protons at 5.5 ppm and 8.70 ppm, respectively (Figure 4a). The obtained polymer has similar absorption characteristic to bare anthracene (Figure 4b). The fluorescence spectrum of diluted solution of **PCL-Anth** in THF excited at λ_exc_ = 350 nm showed the characteristic emission bands of the excited (singlet) anthracene at 595, 655, and 725 nm (Figure 4c). These observations clearly confirmed the successful chain-end functionalization.

**Scheme 2:**
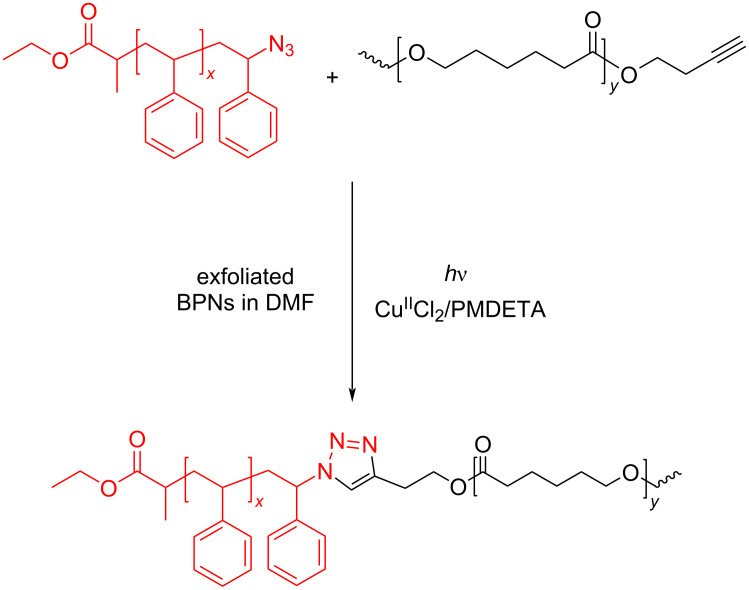
Synthesis of **PS-*b*-PCL** block copolymer via exfoliated BPNs-mediated photoinduced CuAAC reaction.

In addition, block copolymer formation via NIR activated CuAAC process between the polymers having antagonist click components, namely, polystyrene azide (**PS-Az**) and **PCL-Alk**, was investigated. At the end of irradiation in the presence of exfoliated BPNs and Cu^II^Cl_2_/PMDETA, polystyrene-*b*-poly(ε-caprolactone) (**PS-*b*-PCL**) is selectively formed (Scheme 2).

**Figure 5:**
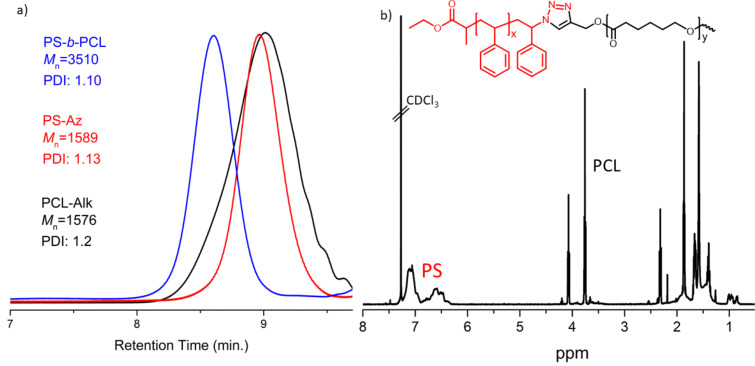
a) GPC traces of **PS-Az**, **PCL-Alk** and block copolymer (**Ps-*b*-PCL**) b) ^1^H NMR spectrum of the block copolymer (**Ps-*b*-PCL**).

Figure 5a displays the GPC traces of precursors **PS-Az**, **PCL-Alk**, and the block copolymer **PS-*b*-PCL**. As it can be seen, the trace of **Ps-*b*-PCL** block copolymer was clearly shifted to higher molecular weight region without contamination of the precursor polymers. The ^1^H NMR spectrum of the block copolymer displayed the characteristic peaks of both macromolecular segments. Additionally, the methylene protons adjacent to the triazole ring at 7.48 ppm were noted (Figure 5b). These results indicated that structurally diverse polymers formed by different polymerization mechanisms can readily be linked just by a simple NIR-induced CuAAC reaction.

**Scheme 3:**
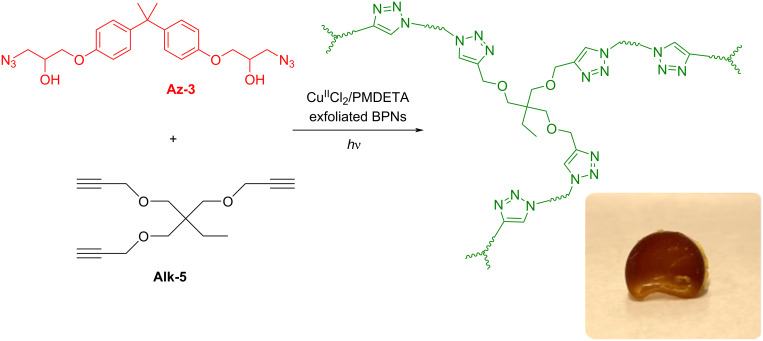
Preparation of the cross-linked polymer by CuAAC reaction using multifunctional monomers, **Az-3** and **Alk-5**.

The macromolecular scope was further extended to the preparation of cross-linked materials. Thus, the formulations containing bisphenol A di(3-azido-2-hydroxypropan-1-ol) ether (**Az-3**), and 1-(prop-2-yn-1-yloxy)-2,2-bis((prop-2-yn-1-yloxy)methyl)butane (**Alk-5**) as multifunctional click components were irradiated in the presence of BPNs and Cu^II^ ligand under NIR light. The gelation was completed after 24 h (Scheme 3).

**Figure 6:**
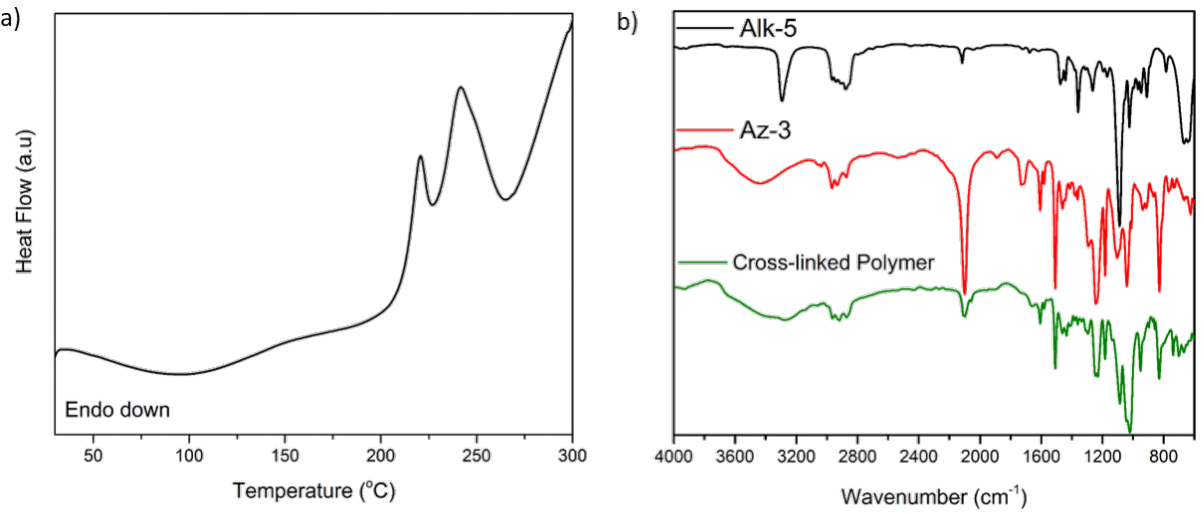
a) DSC thermogram of photoinduced synthesis of nanocomposite networks (heating rate: 10 °C/min). b) FTIR spectra of **Alk-5**, **Az-3** monomers and the corresponding cross-linked polymer.

The photocuring process was also followed by differential scanning calorimetry (DSC). The DSC thermogram shows two exothermic peaks at 220.38 and 241.74 °C, corresponding to the photo click cure reaction in two stages (Figure 6a). Since a complete reaction of all the azide groups could not occur during the dynamic ramping of temperature, the residual azide groups decomposed at higher temperature. The IR spectrum of the cross-linked polymer further demonstrates the formation of a triazole ring by the decrease of the azide peak at 2100 cm^−1^ (Figure 6b).

**Figure 7:**
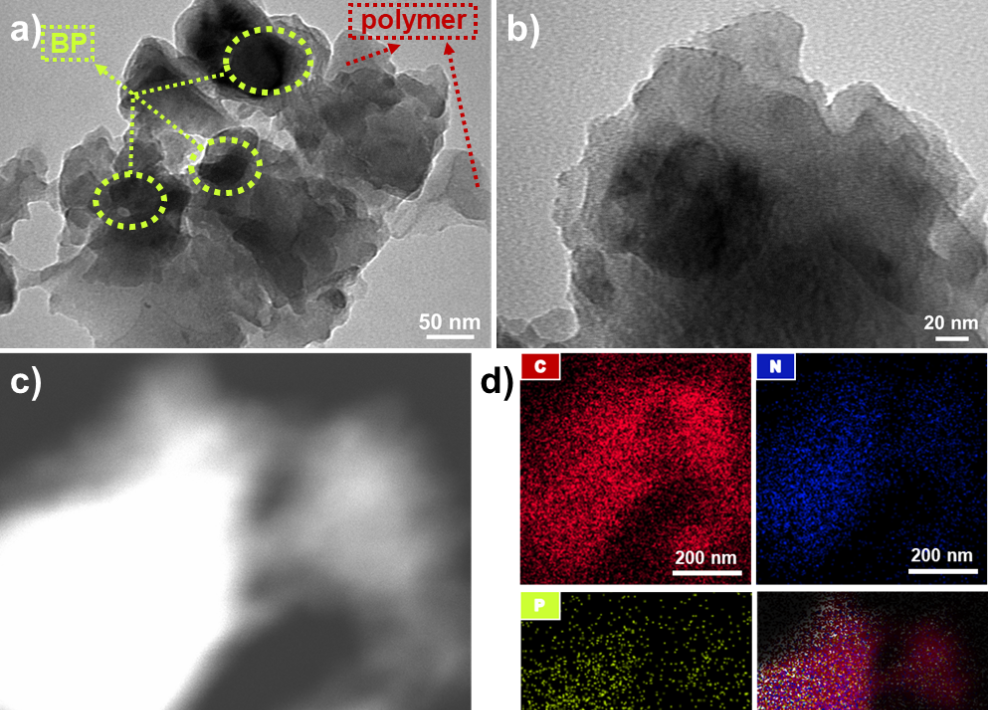
(a, b) TEM images of cross-linked polymer at two different magnifications, c) HAADF-STEM image and d) the associated EDS elemental mapping images of the cross-linked polymer.

Representative TEM images recorded at different magnifications of the resulting cross-linked polymer are shown in Figure 7. From the TEM images, it can be concluded that the process leads to the formation of BPNs-embedded cross-linked polymers. The darker regions circled with yellow dashed line in Figure 6a were attributed to the BPNs while the other relatively lighter regions were ascribed to the cross-linked polymer. To further prove the existence of BPNs in the cross-linked structure, a high-angle annular dark-field scanning TEM (HAADF-STEM) image and the associated elemental mapping images for C, N, and P were recorded and depicted in Figure 7c and 7d. The elemental mapping images adequately demonstrated the presence and the distribution of P atoms that are attributed to BPNs in the cross-linked polymer in addition to C and N atoms (Figure 7d). In contrast to the cross-linked polymer, the distribution of BPNs in the block copolymer structure could not be visualized by TEM, HAADF-STEM, and elemental mapping images (Supporting Information File 1, Figures S5 and S6). This behavior is expected since BPNs are immobilized between the interconnected chains in the cross-linked structure.

## Conclusion

In conclusion, we have demonstrated the use of BPNs as an efficient photoinitiator for the photoinduced CuAAC reactions under white LED and NIR light irradiation. The described method is applicable to organic and macromolecular syntheses. NIR irradiation appeared to be more efficient compared to the while LED due to the higher penetration in the dispersed media. In macromolecular syntheses, polymer chain-end functionalization, block copolymer formation of structurally different polymers and cross-linking polymerization can successfully be achieved by using suitably selected click components. This new method would dramatically extend the applications of photoinduced CuAAC reactions, particularly when the components are light sensitive at short wavelength region and spatial control is required.

## Experimental

### Materials

Red phosphorus (98.9%), tin (99.5%), and tin(IV) iodide (95%) were purchased from Alfa Aesar. Ethyl alcohol (absolute) was obtained from Sigma-Aldrich. Dimethyl sulfoxide (DMSO) was purchased from Merck. All chemicals and solvents were used as received without further purification for synthesis of black phosphorus. Benzyl bromide (Merck), phenylacetylene (Sigma), propargylamine (Sigma), propargyl alcohol (Sigma), *d*-dimethyl sulfoxide, (DMSO-*d*_6_, Merck), *N*,*N*,*N*’,*N*’’,*N*’’-pentamethyldiethylenetriamine (PMDETA, Aldrich), sodium azide (NaN_3_, Panreac), copper(II) chloride (Cu^II^Cl_2_, Merck), black phosphorus, dimethyl sulfoxide (DMSO) was used as received. Propargyl acrylate (Sigma), styrene (Merck) were purified before by using a basic alumina column to remove the inhibitor and then stored in the fridge. ε-Caprolactone (Merck), and stannous octoate (Aldrich) were dried with CaH_2_ under vacuum.

### Characterizations

^1^H NMR spectra were recorded at room temperature at 500 MHz on an Agilent VNMRS 500 spectrometer. Gel permeation chromatography (GPC) measurements were performed on a TOSOH EcoSEC GPC system equipped with an auto sampler system, a temperature-controlled pump, a column oven, a refractive index (RI) detector, a purge and degasser unit and a TSKgel superhZ2000, 4.6 mm ID × 15 cm × 2cm column. Tetrahydrofuran was used as an eluent at a ﬂow rate of 1.0 mL/min at 40 °C. The refractive index detector was calibrated with polystyrene standards having narrow molecular-weight distributions. The data were analyzed using Eco-SEC analysis software. A Hitachi HT7700 (TEM) with EXALENS (120 kV) working at a high-resolution (HR) mode was used to obtain transmission electron microscopy (TEM) images, high-angle annular dark field (HAADF) scanning transmission microscope (STEM) images and the associated EDS elemental mapping images.

### Synthesis of black phosphorus crystals and preparation of its nanosheets

Black phosphorus (BP) was prepared using a modified low-pressure chemical vapor transport method [40,42–43]. For the synthesis, 500 mg of red phosphorus, 20 mg of Sn and 10 mg of SnI_4_ were placed into a quartz ampoule with the dimensions of 20 cm length and 1.5 cm width. The air was evacuated by vacuum, and the ampoule was left to dry at least for 30 min under vacuum. The sealed ampoule was placed horizontally in a muffle furnace. The applied heating program was as follows: firstly, the temperature raised to 893 K in 5 h and kept at this temperature for 5 h. Next, the temperature was lowered to 758 K in the span of 6 h and the temperature was kept at this temperature for 2 h. Finally, the oven was cooled to 393 K in 5 h, and it was left for natural cooling afterwards. After the heating process, the ampoule was cracked in dry toluene and the crystalline BP was separated. In order to remove surface impurities, the BP crystals were transferred into absolute ethanol and sonicated for 30 minutes. The sonicated crystals were carefully transferred to a Schlenk tube and dried under vacuum. The Schlenk tube was filled with argon and crushed under inert atmosphere. The produced BP crystals were stored under vacuum.

BP nanosheets were prepared by the liquid phase exfoliation of BP crystals. A specific amount of BP was dispersed thoroughly in DMSO by a sonication bath (200 W) for 10 h at 6 °C. The resulting BP nanosheets dispersion was kept under an inert atmosphere for the further use.

### Preparation of azide and alkyne derivatives

#### Synthesis of benzyl azide (**Az-1**)

A literature procedure was used [44]. Product was obtained pale yellow oil, yield 96%. ^1^H NMR (500 MHz, DMSO-*d*_6_) δ 7.43–7.34 (m, 5H, -C_6_H_5_), 4.43 (s, 2H, CH_2_-N_3_). FTIR: 2108 cm^−1^.

#### Synthesis of (azidomethyl)anthracene (**Az-2**)

A literature procedure was used [45]. 9-Hydroxymethylanthracene (7.40 mmol, 1 equiv) was added to DCM (50 mL) and cooled to 0 °C. Then, SOCl_2_ (1.5 equiv) was slowly introduced to the reaction media and allowed to warm up to room temperature while being stirred for 1 h. The solvent was removed under vacuum and the residue redissolved in DMF (10 mL). Following dissolution of the compound, NaN_3_ (1.5 equiv) was added, and the reaction was stirred at 50 °C. After 1 h, the reaction mixture was allowed to cool down, diluted with water and extracted with EtOAc. The combined organic phases were washed with brine, dried with anhydrous MgSO_4_, filtered, and concentrated under vacuum. Brownish yellow crystalline solid, yield = 93%. ^1^H NMR (500 MHz, DMSO-*d*_6_) δ 8.70 (s, 1H), 8.44 (dd, 2H), 8.14 (dd, 2H), 7.64 (td, 2H), 7.56 (td, 2H), 5.51 (s, 2H); ^13^C{^1^H} NMR (DMSO-*d*_6_, 125 MHz) δ 131.39, 130.72, 129.51, 129.06, 127.28, 126.96, 125.88, 124.51, 45.96.

#### Synthesis of bisphenol A di(3-azido-2-hydroxypropan-1-ol) ether (**Az-3**)

Diazido monomer, bisphenol A di(3-azido-2-hydroxypropan-1-ol) (**Az-3**) was synthesized according to a described method [46]. **Az-3** was obtained as light yellowish viscous oil and was directly used without further purification, yield 98%. ^1^H NMR (500 MHz, CDCl_3_) δ 7.15 (m, 4H), 6.82 (m, 4H), 4.16 (m, 2H), 4.0 (dd, 4H), 3.51 (m, 4H), 1.65 (s, 6H); ^13^C{^1^H} NMR (CDCl_3_, 125 MHz) δ 131.38, 130.67, 129.51, 129.09, 127.26, 126.83, 125.80, 45.93.

#### Synthesis of 1-(prop-2-yn-1-yloxy)-2,2-bis((prop-2-yn-1yloxy)methyl)butane (**Alk-5**)

A literature procedure was followed [47]. The crude obtained product was then purified using column chromatography to give a clear oil, yield 70%. ^1^H NMR (500 MHz, DMSO-*d*_6_) δ 0.80 (t, 3H, CH_3_), 1.30 (q, 2H, CH_2_-CH_3_), 3.30 (s, 6H, CH_2_), 4.10 (d, 6H, CH_2_-alkyne); ^13^C{^1^H} NMR (DMSO-*d*_6_, 125 MHz) δ 7.84 (1C, CH_3_), 23.33 (1C, CH_2_), 42.82 (1C, C), 58.39 (3C, CH_2_-alkyne), 70.13 (3C, CH_2_), 77.24 (3C, CH_2_), 80.83 (3C, C, alkyne).

#### Synthesis of ω-azido terminated polystyrene (**PS-Az**)

ω-Bromo functional polystyrene was synthesized by ATRP according to a reported procedure [48]. In a flask equipped with a magnetic stirrer, PS-Br (1 equiv) and sodium azide (5 equiv) were dissolved in 5 mL DMF. The reaction mixture was stirred at room temperature 24 h, then precipitated in 10-fold excess of methanol, filtered and dried in vacuum to yield PS-N_3_. Yield 95% (*M*_n,GPC_: 1589 g·mol^−1^, *M*_w_/*M*_n_: 1.13). FTIR: 2096 cm^−1^.

#### Synthesis of acetylene-terminated poly(ε-caprolactone) (**PCL-Alk**)

Acetylene-terminated PCL-Alk was synthesized according to a modified procedure [49]. To a Schlenk tube, 3-butyn-1-ol was dissolved in ε-caprolactone and heated to 110 °C under nitrogen. After the reaction mixture warmed up homogeneously, one drop of tin octoate was added to the reaction media and the solution was stirred for 3 hours. The obtained polymer was dissolved in chloroform and precipitated in methanol:water (2:1) to yield poly(ε-caprolactone). White solid, (85%) *M*_n,GPC_: 1576 g·mol^−1^, *M*_w_/*M*_n_: 1.2. FTIR: 2102 cm^−1^.

### Photoinduced CuAAC reactions

#### Synthesis of organic molecules

For the first step of the reaction an appropriate amount of black phosphorus was exfoliated in DMSO-*d*_6._ In a typical experiment, exfoliated BP in DMSO-*d*_6_ (0.5 mL) and azide compound (1 mmol, 1 equiv) were added to a NMR tube containing Cu^(II)^Cl_2_ (0.05 equiv), PMDETA (0.1 equiv). After 5 min, alkyne derivative (1 mmol, 1 equiv) was added slowly to the NMR tube. The reaction tube was irradiated by using a Philips 150 W PAR38E E27 halogen pressure glass type bulb with strong IR-A (NIR) emission. The light intensity inside the reaction tube was ≈200 mW·cm^−2^. The light bulb was attached to the top of a photoreactor setup equipped with a large air cooling fan and the reaction temperature was kept constant at room temperature (24−25 °C). ^1^H NMR spectra were recorded 4 h later.

#### Synthesis of anthracene functional poly(ε-caprolactone) (**PCL-Anth**)

The same process as in the block copolymerization was applied. **Az-2** (19.27 mg, 1 equiv), **PCL-Alk** (1 equiv), CuCl_2_ (1 equiv) and PMDETA (1 equiv) were placed in a Schlenk tube. The tube was degassed by three freeze pump-thaw cycles. Then the tube was irradiated under NIR light for 48 h. After the given time, the mixture was diluted with THF and the copper complex was removed by passing through a neutral alumina column. Excess amount of THF was evaporated by a rotary evaporator. After precipitation of the mixture to cold methanol, the polymer was collected by filtration and dried under vacuum overnight. ^1^H NMR was demonstrated in Figure 4.

#### Synthesis of polystyrene-*b*-poly(ε-caprolactone) (**PS-*b*-PCL**)

Firstly, under dark conditions BP was exfoliated in dry DMF by a sonic bath for 8 h at 10 °C. Subsequently, the solution was transferred into a centrifuge at 2500 rpm for 15 min. Terminally, this exfoliated BPNs with **PS-Az** (200 mg, 1 equiv), Cu^II^Cl_2_ (1 equiv), PMDETA (1 equiv) and **PCL-Alk** (1 equiv) were placed in a Schlenk tube. The tube was degassed by three freez-pump thaw cycles. Then the tube was irradiated with NIR light 48 h. At the end of the reaction, the mixture diluted THF and the copper complex was removed by passing it through a neutral alumina column. Excess amount of THF was evaporated by a rotary evaporator. After precipitation of the mixture to cold methanol, the polymer was collected by filtration and dried under vacuum overnight. *M*_n_,_GPC_: 3510 g·mol^−1^, *M*_w_/*M*_n_: 1.10.

#### Synthesis of cross-linked polymer

**Az-3** and **Alk-5** was mixed in equal ratio (1 equiv) with Cu^II^Cl_2_ (0.05 equiv) and PMDETA (0.1 equiv) in a small transparent vial and 300 µL BPNs in DMF was added to the vial, then irradiated 4 h. After the gelation was completed, the gel was placed in DCM for 24 h hours, then filtered and dried 24 h in a vacuum oven.

## Supporting Information

File 1 Characterisation data: ^1^H NMR spectra of **Alk-1**, **Alk-2** and **Alk-3**, TEM, HAADF-STEM and associated EDS elemental mapping of **PS-*b*-PCL**.
