# Supplementary material for: Exfoliated black phosphorous-mediated CuAAC chemistry for organic and macromolecular synthesis under white LED and near-IR irradiation
Source: Beilstein J Org Chem. 2021 Sep 23;17:2477–87. doi: 10.3762/bjoc.17.164 (PMC8474068; doi:10.3762/bjoc.17.164)
Supplement: File 1 — Characterisation data: 1H NMR spectra of Alk-1, Alk-2 and Alk-3, TEM, HAADF-STEM and associated EDS elemental mapping of PS-b-PCL. [file Beilstein_J_Org_Chem-17-2477-s001.pdf]

BEILSTEIN JOURNAL OF ORGANIC CHEMISTRY

Supporting Information

for

Exfoliated black phosphorous-mediated CuAAC chemistry for organic and macromolecular synthesis under white LED and near-IR irradiation

Azra Kocaarslan, Zafer Eroglu, Önder Metin and Yusuf Yagci

Beilstein J. Org. Chem. **2021,** *17,* 2477–2487. doi:10.3762/bjoc.17.164

Characterisation data: ^{1}H NMR spectra of Alk-1, Alk-2 and Alk-3, TEM, HAADF-STEM and associated EDS elemental mapping of PS-*b*-PCL

License and Terms: This is a supporting information file under the terms of the Creative Commons Attribution License (https://creativecommons.org/licenses/by/4.0). Please note that the reuse, redistribution and reproduction in particular requires that the author(s) and source are credited and that individual graphics may be subject to special legal provisions.
The license is subject to the *Beilstein Journal of Organic Chemistry* terms and conditions: (https://www.beilstein-journals.org/bjoc/terms)

CONTENTS

Figure S1. ^{1}H NMR spectra of the reaction between benzyl azide and propargyl acrylate after exposure using BPNs under NIR light irradiation.

Figure S2. ^{1}H NMR spectra of the reaction between benzyl azide and propargyl amine after exposure using exfoliated BP under NIR light irradiation.

Figure S3. ^{1}H NMR spectra of the reaction between benzyl azide and propargyl alcohol after exposure using exfoliated BP under NIR light irradiation.

Figure S4. Kinetic study of CuAAC reaction between propargyl alcohol and benzyl azide under NIR light.

Figure S5. TEM images of **a** and **b** PS-*b*-PCL block copolymer at different magnification.

Figure S6. HAADF-STEM image **a** associated EDS elemental mapping images **b** of PS-*b*-PCL block copolymer.

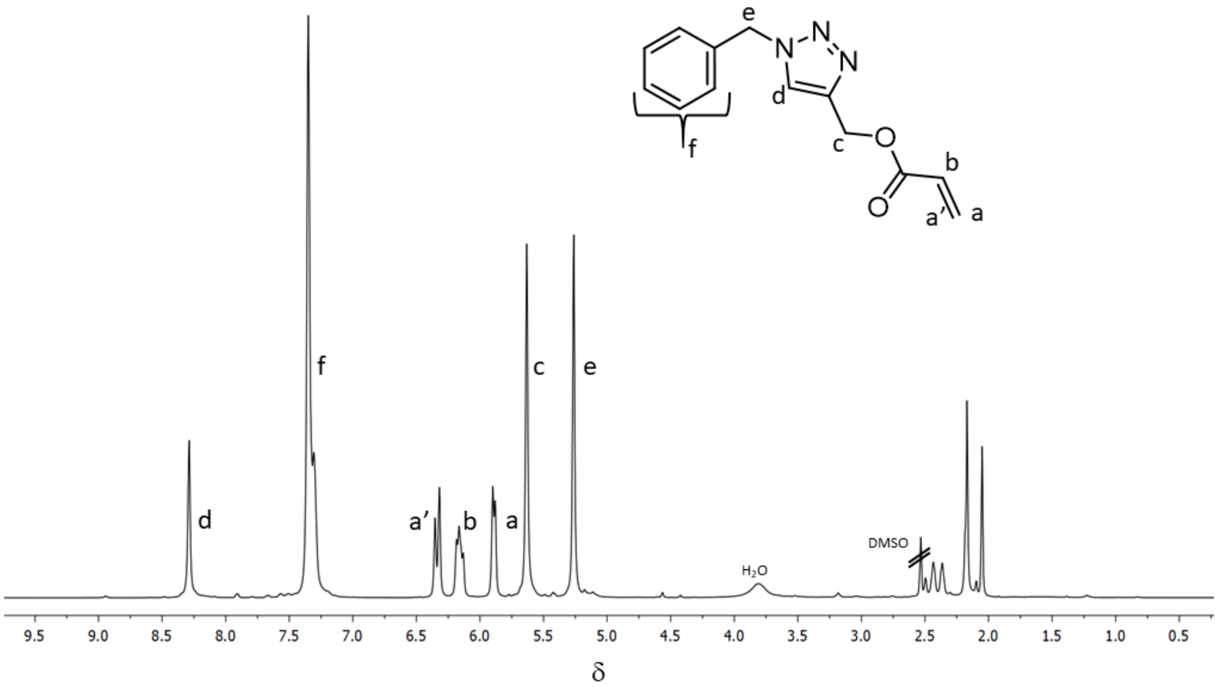

Figure S1. ^{1}H NMR spectra of the reaction between benzyl azide and propargyl acrylate after exposure using BPNs under NIR light irradiation.

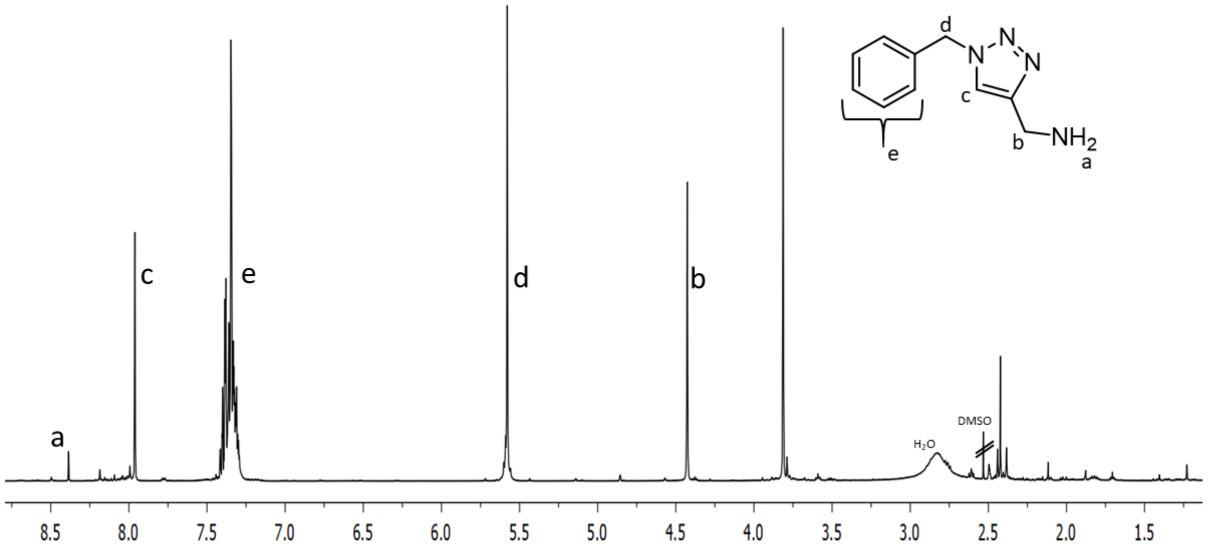

Figure S2. ^{1}H NMR spectra of the reaction between benzyl azide and propargyl amine after exposure using exfoliated BP under NIR light irradiation.

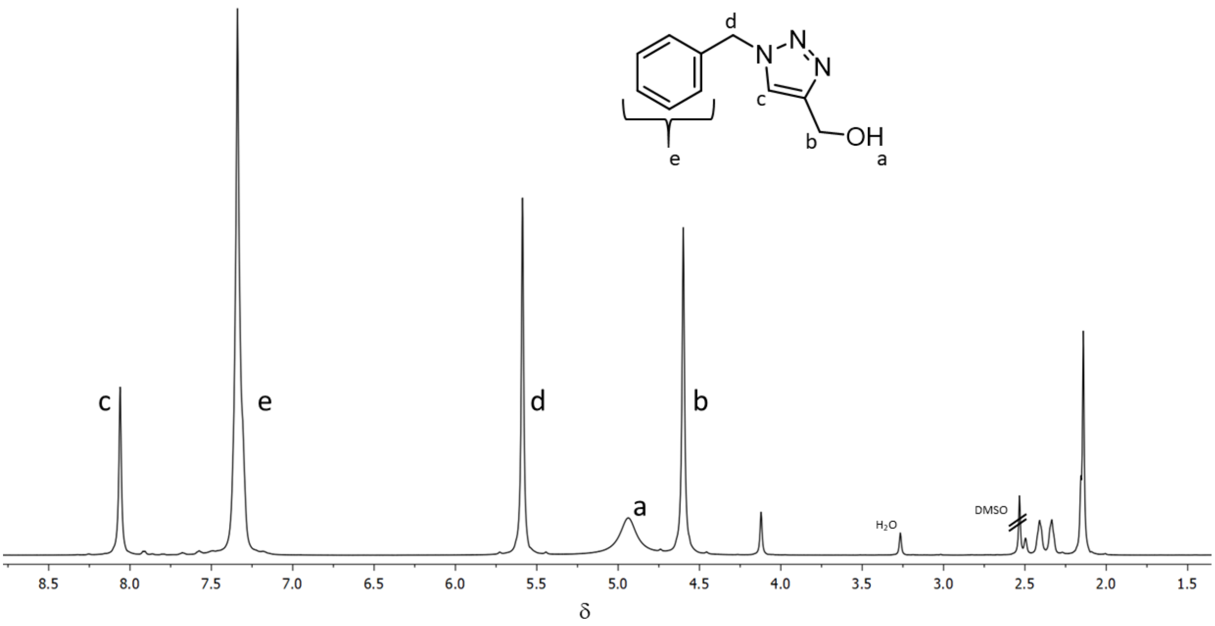

Figure S3. ^{1}H NMR spectra of the reaction between benzyl azide and propargyl alcohol after exposure using exfoliated BP under NIR light irradiation.

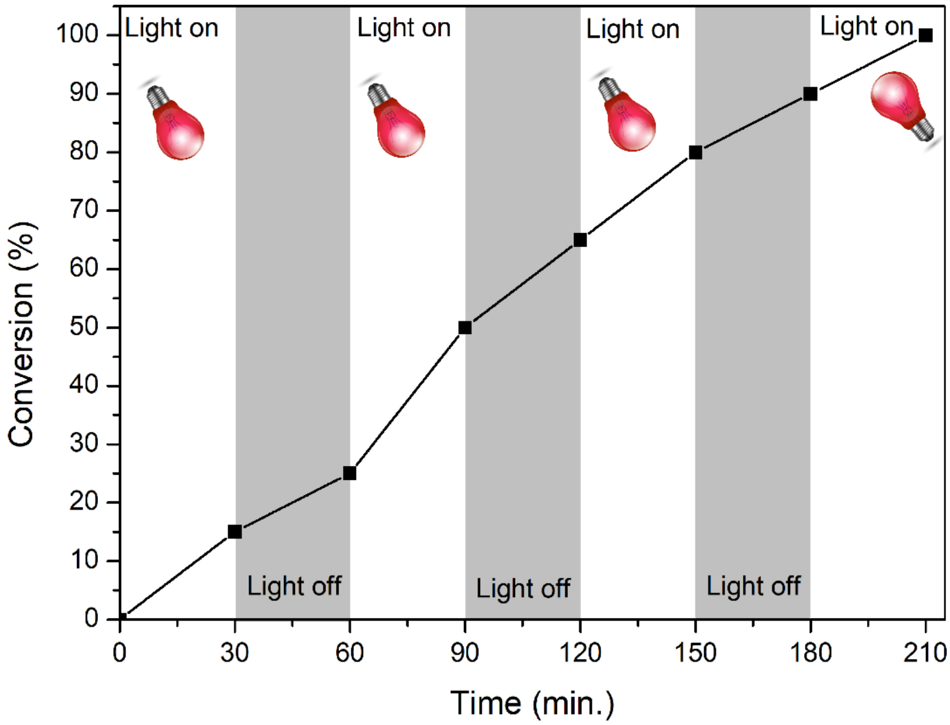

Figure S4. Kinetic study of CuAAC reaction between propargyl alcohol and benzyl azide under NIR light.

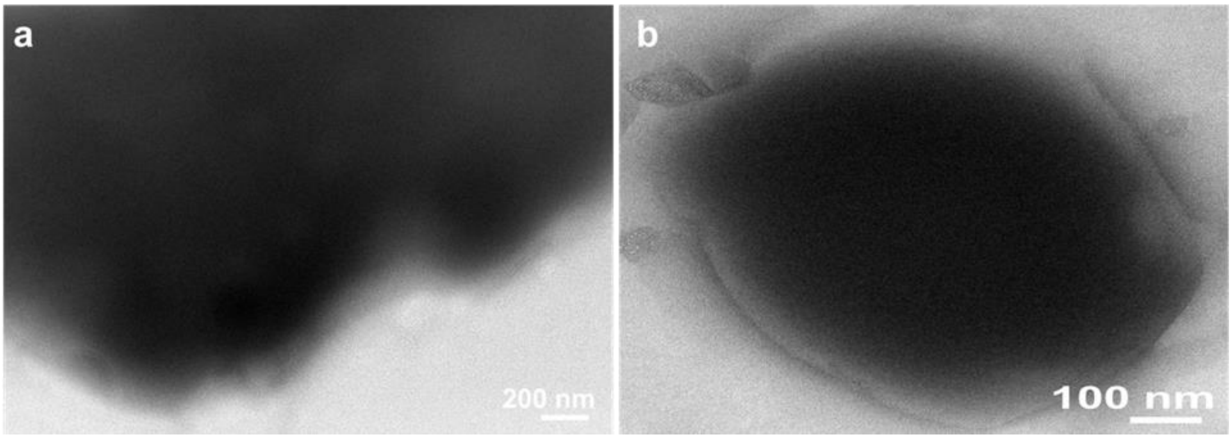

Figure S5. TEM images of **a** and **b** PS-*b*-PCL block copolymer at different magnification.

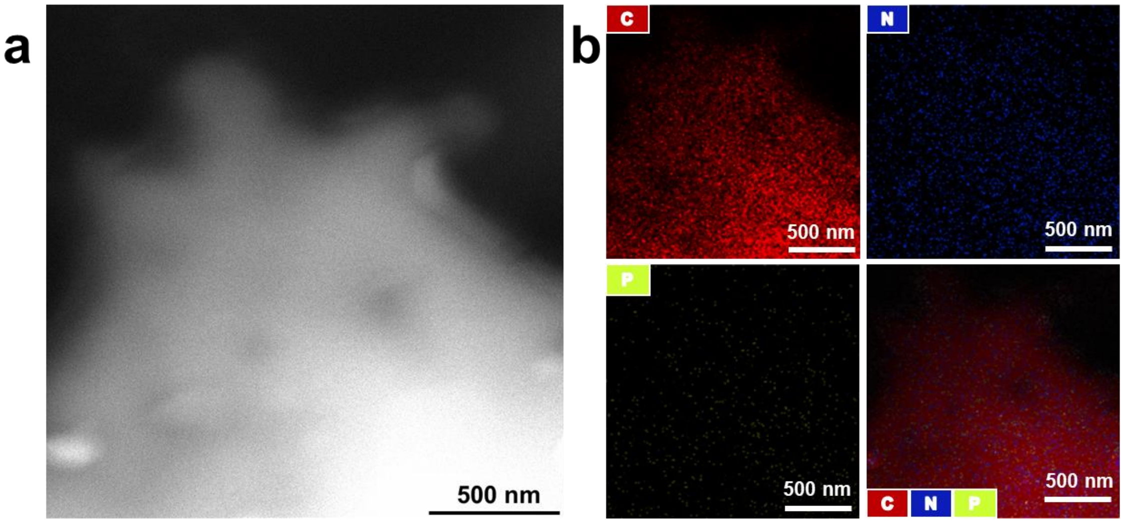

Figure S6. HAADF-STEM image **a** associated EDS elemental mapping images **b** of PS-*b*-PCL block copolymer.
